# Evaluation of effectiveness resistance genes in wheat genotypes using marker-assisted selection for stripe rust resistance breeding”

**DOI:** 10.1186/s12870-024-05470-1

**Published:** 2024-08-20

**Authors:** Atef A. Shahin, Reda I. Omara, Hend A. Omar, Heba Saad El-Din, Mohamed D. Sehsah, Tarek Essa, Marwa A. Zayton, Hanaa S. Omar

**Affiliations:** 1 Plant Pathology Research Institute, Agricultural Research Center (ARC), Giza, 12619 Egypt; 2 Department of Plant Pathology, Faculty of Agriculture, Cairo University, Giza, 12613 Egypt; 3 Department of Genetics, Faculty of Agriculture, Cairo University, PO, Giza, 12613 Egypt

**Keywords:** Breeding programs, Effective *yr* genes, Stripe rust, Slow rusting resistance, Wheat

## Abstract

Stripe rust, induced by *Puccinia striiformis* f. sp. *tritici*, is the most harmful and prevalent disease in temperate regions worldwide, affecting wheat production areas globally. An effective strategy for controlling the disease involves enhancing genetic resistance against stripe rust, achieved through Egyptian breeding efforts not previously conducted on wheat genotypes. The resistance level to stripe rust in thirty-eight wheat genotypes was assessed using marker-assisted selection methods. The investigation suggests that wheat breeding programs can utilize slow-rusting Yr genes, which are effective resistance genes, to develop novel genotypes with stripe rust resistance through marker-assisted breeding. Based on the four disease responses of the wheat genotypes under investigation, the results categorized the genotypes into three groups. The first group included resistant genotypes, the second group exhibited a slow-rusting character with the lowest disease symptom rates, and the last group displayed the highest disease characteristics rates throughout the three seasons, comprising fast-rusting genotypes. The rust-resistant genes identified were *Yr5*,* Yr9*,* Yr10*,* Yr15*,* Yr17*,* Yr18*,* Yr26*,* Yr29*,* Yr30*, and *Yr36*. Genes *Yr26*,* Yr30*,* and Yr36* were present in all genotypes. Genotypes Misr3, Misr4, Giza168, Giza167, Giza170, Giza171, Gemmeiza9, and Gemmeiza10 carried the *Yr9* gene. Only one genotype, Sids13, was found to have the *Yr17* gene. Genes *Yr18* and *Yr29* were identified in Sids14, Giza168, Giza170, Gemmeiza9, and Gemmeiza10. However, none of the wheat genotypes showed the presence of *Yr5*,* Yr10*, or *Yr15*. Several backcrossing generations were conducted to introduce the *Yr5* and *Yr10* genes into susceptible genotypes (Misr1, Misr2, and Gemmeiza11). These genotypes are cultivated globally and are known for producing high-quality flour, making them of great importance to farmers. The study demonstrates significant potential for enhancing wheat genotypes for stripe rust resistance and increased production.

## Introduction

Wheat (*Triticum aestivum*) is one of the most strategically and commercially significant cereal crops in the world [1]. Rust diseases are a serious hindrance to wheat production, and the alarming gap between the growing demand and supply of wheat is concerning. Stripe rust is one of these dangerous rust diseases. It is a terrible fungal disease that affects wheat [2]. Wheat stripe rust was first discovered in Lower Egypt [3]. Recently, the highest grain yield losses were recorded on the wheat cultivar Gemmeiza11 (64.20%), followed by Misr1 (62.38%), Misr2, (57.66%) and Sids12 (50.89%) in the North Delta region of Egypt [4]. The development of resistant genotypes is an efficient way to control wheat stripe rust; however, this requires information on the pathogen population, pathogenicity diversity, and race distribution in specific places and times, in addition to knowledge of the resistance genes that are effective against these races [5]. To prevent crop losses, it’s also critical to predict changes in population pathogenicity and develop a rust control plan [6]. For wheat breeders worldwide, improving resistance against wheat stripe rust is one of the major difficulties in this regard. Breeding and developing resistant wheat cultivars are the most economical and sustainable way to avoid stripe rust. More than sixty genes, including *Yr5*,* Yr15*,* Yr37*, *Yr51* and *Yr57*, regulate resistance to yellow rust have been mapped on different wheat chromosomes.

Quantitative loci (QTLs) and several temporarily designated resistance genes can also offer partisan or comprehensive protection against a variety of rust pathotypes [7]. To offer long-lasting resistance, more resistance genes are needed to diversify the gene pyramid combinations used to provide stripe rust resistance. A sustainable and environmentally friendly method of managing diseases is to create wheat genotypes with effective disease-resistance genes. To create rust-resistant wheat genotypes, a multitude of rust-resistance sources (genes) were found in the worldwide genotypes or related species of wheat [8]. Rust isolates with accessible pathogenicity are used to postulate resistance genes [9]. Occasionally only molecular markers can be used to identify resistance genes [10]. One of the most crucial strategies for preventing wheat rusts is genetic resistance since it is the first line of defense, an effective and affordable control measure, and it doesn’t add to the farmer’s expenses. However, in the past, only significant gene-based resistance was employed [11, 12]. Moreover, only a few significant genes contributed to the majority of the wheat genotypes that have become available. Major gene-based resistance is quickly lost due to the stripe rust pathogen’s ability to rapidly acquire new virulence to resistance genes and pathogenic diversity induced by mutation, migration, and somatic or sexual recombination [13, 14]. Such the most important gene-based resistance is speedily lost. Under this collective, resistance to numerous races of the pathogen is essential. Durable resistance is a variety of non-specific resistance that fights a variety of pathogen races [15].

Several efficient markers for genes resistant to stripe rust have been developed in the last fifteen years. However, many wheat genotypes have lost their resistance, and some of the genes responsible for resistance have not been identified. Therefore, the current study aims to assess the level of slow-rusting resistance in thirty-eight wheat genotypes under field conditions. Additionally, the study seeks to identify the stripe rust resistance genes (Yr’s) using molecular markers. Furthermore, efforts were made to introduce the resistant genes into susceptible wheat genotypes to highlight the importance of marker-assisted backcross breeding in wheat.

## Materials and methods

### Plant materials

The experimental material, which includes the plant material supplied, complies with institutional, national, and international guidelines and legislation. Thirty-eight wheat genotypes (*Triticum aestivum*) were provided by the Wheat Research Department, Field Crops Institute, Egypt, and included resistance genes that were not known to exist (Table 1). These local varieties were obtained following scientific guidelines and intellectual property standards from the Field Crops Research Institute at the Agricultural Research Center in Egypt. At the Sakha Agriculture Research Station in Egypt, genotypes were assessed for stripe rust resistance at the adult plant stage through artificial inoculation. Furthermore, the International Maize and Wheat Improvement Center (CIMMYT), Mexico, provided fifty-two yellow rust differential sets and isogenic lines with recognized stripe rust resistance genes (*Yr’s*) and Morocco (check very vulnerable), which were utilized and assessed in this study (Table 2).

**Table 1:** The thirty-eight wheat genotypes and their pedigrees utilized in this investigation

No.	Genotypes	Pedigree
1	Misr1	OASIS/SKAUZ//4*BCN/3/2*PASTOR
2	Misr2	SKAUZ/BAV92
3	Misr3	ATTILA*2/PBW65*2/KACHU
4	Misr4	NS-732/HER/3/PRL/SARA/TSI/VEE#5/4/FRET2/5/Wheat/SOKOLL
5	Sakha8	INDUS/NORTENO"S”-PK348
6	Sakha61	INIA–RL4220//7c/Yr “S” CM15430-25-55-0 S-OS
7	Sakha69	INIA/RL 4220//7c/Yr “S” CM 15430-25-65–0 S-0 S
8	Sakha62	INIA–RL4220//7c/Yr “S”
9	Sakha92	NAPO 63/ INIA 66 // WERN “S”
10	Sakha93	Sakha 92/TR 810,328 S 8871-1–2 S-1 S–0 S
11	Sakha94	OPATA/RAYON//KAUZ CMBW9043180-OTOPM-3Y-010 M…
12	Sakha95	PASTOR//SITE/MO/3/CHEN/AEGILOPS/SQUARROSA(TAUS)// …
13	Gemmeiza1	Maya74 / On-1//1160 − 147/3/Bb/Gallo/4/chat “S”
14	Gemmeiza3	BB/7 C*//Y50/KAL*3× Sakha 8/4/ PRV/WW/5/BJI “S”//ON*3/BON
15	Gemmeiza5	VEE”S”/SMW6525 Gm.4017-1Gm.7Gm.-3Gm.-0Gm
16	Gemmeiza9	Ald “S”/Huas//CMH74A.630/Sx CG4583-5G-1G-0G
17	Gemmeiza10	MAYA 74 “S”/ON//1160 − 147/3/BB/GLL/4/CHAT"S”/5/CROW “S”
18	Gemmeiza11	BOW’’S’’/KVZ’’S’’//7 C/SERI82/3/GIZA168/SKHA61
19	Giza150	MIDA-CADET/2* GIZA139
20	Giza155	REGENT/2*GIZA139// MIDA-CADET/2* HINDI62
21	Giza157	(Regent 975 − 11 × Giza 1392) × Mida Cadet × Hindi 62
22	Giza160	CHENAB/GIZA155
23	Giza162	VCM//CN 067/7 C/3/Kal/Bb. CM8399-D-4 M-3Y-1 M-1Y-1 M-0Y
24	Giza 163	T. aestivum/ Bon // CNO / 7 C.
25	Giza164	KVZ / Buha “S” // Kal / Bb.
26	Giza165	CNO/MFD//MON “S”.
27	Giza167	AU/UP301//GLL/SX/B/Pew”S”/4/Mai”S”/Maya”S”//Pew”S”.
28	Giza168	MRL/BUC//SERI. CM93046-8 M-0Y-0 M-2Y-0B-0SH
29	Giza170	MIL/BUC//Seri CM93046-8 M-0Y-0 M-2Y-0B
30	Giza171	Sakha93/Gemmeiza9 S 6-1GZ-2GZ-2GZ-0 S
31	Sids1	HD2172/Pavon‘‘S’’//1158.57/Maya74"S”. SD46-4Sd-2SD-1SD-0SD
32	Sids4	Maya"S”/MON"S”// CMH74A.2/3/*2 Giza157.
33	Sids6	Maya “S”/Mon‘‘S’’//CMH174A.592/3/Sakha8*2
34	Sids8	Maya “S”/Mon‘‘S’’//CMH74A.592/3/Sakha8*2
35	Sids12	Buc//7c/ald/5/maya74/on//1160 − 147/3/bb/gll/4/chat’’s’”
36	Sids13	AMAZ19 = KAUZ"S”//TSI/SNB"S”
37	Sids14	Bow’’s’’/Vee’’s’’//Bow’s’/Tsi/3/BANI SUEF 1 SD293-1SD-2SD-4SD-0SD
38	Shandweel1	SITE/MO/4/NAC/TH.AC//3*PVN/3/MIRLO/BUC

**Table 2:** Fifty-two yellow rust differential sets and isogenic lines (from CIMMYT) used in this study

No	Name	No	Name
1	Morocco	27	AOC-YR*3//LALBMONO1*4/PVN
2	Avocet-YRA	28	AOC-YR*3/PASTOR
3	Avocet + YRA	29	POLLMER
4	YR1/6*AOC	30	PASTOR
5	SIETE CERROS T66	31	REBECA F2000
6	TATARA	32	FRANCOLIN#1
7	YR5/6*AOC	33	AOC-YR/QUAIU#3
8	YR6/6*AOC	34	OPATA/PASTOR
9	YR7/6*AOC	35	OPATA/PASTOR
10	YR8/6*AOC	36	AOC-YR/QUAIU#3
11	YR9/6*AOC	37	M10(MUTATED C-306)/AOC-YR
12	YR10/6*AOC	38	CHUAN NONG 19
13	YR15/6*AOC	39	IRAGI
14	YR17/6*AOC	40	KOELZ W 11,192:AE
15	YR18/3*AOC	41	PBW343/KKU
16	YR24/3*AOC	42	AOC-YR*3//LALBMONO1*4/PVN
17	YR26/3*AOC	43	YR33
18	YR27/6*AOC	44	YR34
19	YRSP/6*AOC	45	YR35 98M71
20	PAVON F 76	46	YR37
21	SERI M82	47	YR4PL
22	OPATA M85	48	YR51
23	SUPER KAUZ	49	YR54
24	YRCV/6*AOC	50	YR57
25	PBW343	51	YRKK
26	AOCYR*3/3ALTAR84/AE.SQ//OPATA	52	YRALd

### Field experiments

The experiments were conducted at the Sakha Agriculture Research Station in the Kafr El-Sheikh Governorate. Kafr El-Sheikh is situated in the northern region of the Nile Delta, within the Rosetta and Damietta Nile branches. The governorate is positioned between 31° 30′ 7.59° and 31° 9′ 58.09° N and between 30° 20′ 36.83° and 31° 17′ 15.16° East. In addition, the experiments were performed at the Plant Pathology Research Institute, Agricultural Research Center (ARC) (30°01’17"N 31°12’36” E) and Faculty of Agriculture, Cairo University, Giza, Egypt )30°01’04"N 31°12’37” E (during the three successive seasons 2021, 2022, and 2023. Thirty-eight genotypes and 52 yellow rust differential sets and isogenic lines were sown in two rows (3 m long and 30 cm apart). Each row was sown with 5 g of a given tested genotype in a randomized complete block design (RCBD), with three replications at the adult plant stage. The experiment was surrounded by a 1 m allay and a 1.5 m belt which served as spreaders of yellow rust-susceptible entry, that is, Morocco, repeatedly between wheat genotypes every 10 lines. The spreader plants were inoculated in the tillering stage using urediniospores mixture in a hypodermic syringe and mixed with talcum powder at a ratio of 1:20 (w: w) and dusted. The pathogen then naturally extends from these primary sources of infection. The genotypes studied were evaluated under greenhouse conditions. Each genotype was grown in plastic pots with a 25 cm diameter, with three replicates in a completely randomized block design. The recommended agricultural practices were applied. Artificially inoculated urediniospores of old races (the *Yr27* race) and Warrior races for each genotype at the tillering stage were examined. The inoculum races used were virulent for *Yr2*,* Yr6*,* Yr7*,* Yr8*,* Yr9*,* Yr25*, and *Yr27*, and avirulent for *Yr5*,* Yr10*, and *Yr15*, as shown in Table (3). The responses of all studied wheat genotypes to the *P. striiformis* pathotypes were noted at the adult plant stage using a modified Cobb’s scale method [15, 16]. In a nursery that was artificially infected, the resistance to field stripe rust (based on near-isogenic lines) of three recurrent parents (i.e., donor parents, backcross plants, and control) to field stripe rust (based on near-isogenic lines) was assessed. The extent of disease development was estimated in terms of host reaction, resistance, susceptibility, and severity. By recreating the intensity of the infection by assigning a constant value for the host response, the average coefficient of infection (ACI) was calculated from these three data points for statistical analysis [17]. Based on their resistance, the lines were categorized as resistant or susceptible to segregation. By recreating the intensity of the infection by assigning a constant value for the host response, the average coefficient of infection (ACI) was calculated from these three data points for use in the statistical analysis [17]. Based on their resistance, the lines were categorized as resistant, and susceptible to segregation.

**Table 3:** Virulence phenotype of old, *Yr27* and warrior races on *yr* genes

	Virulence phenotype																		
Race	*Yr1*	*Yr2*	*Yr3*	*Yr4*	*Yr5*	*Yr6*	*Yr7*	*Yr8*	*Yr9*	*Yr10*	*Yr15*	*Yr17*	*Yr24*	*Yr25*	*Yr27*	*Yr32*	*YrSP*	*AvS*	*YrAmb*
Old race		v				v	v	v	v					v	v			v	
Race Yr27		v				v	v	v	v				v	v	v			v	
Warrior race	v	v	v	v		v	v		v			v		v		v	v	v	v

Figures and symbols designate virulence and a virulence (-) corresponding to yellow rust resistance genes: *Yr1*,* Yr2*,* Yr3*,* Yr4*,* Yr5*,* Yr6*,* Yr7*,* Yr8*,* Yr9*,* Yr10*,* Yr15*,* Yr17*,* Yr24*,* Yr25*,* Yr27*,* Yr32* and the resistance specificity of Spalding Prolific (Sp), AvocetS (AvS) and Ambition (Amb), respectively, *v =* virulent, blank = a virulent (*av*)

### Disease assessment

Disease severity was assessed using the modified Cobb Scale [18] as infection types, where 0 = no visible infection, R = resistant (necrotic areas with or without small pustules), MR = moderately resistant (small pustules surrounded by necrotic areas), MS = moderately susceptible (medium-sized pustules, no necrosis, but some chlorosis possible), and S = susceptible (large pustules, no necrosis or chlorosis). Disease severity (0–100%) was recorded based on the estimated percentage of leaf area covered by lesions (0% = no visible infection; 100% = complete leaf coverage). Subsequently, the coefficient of infection (CI) was calculated by multiplying the response value by the disease severity (%). The average coefficient of infection (ACI) was derived from the sum of CI values for each entry (Table 4).

**Table 4:** The observation on wheat stripe rust response

Reaction	Observation	Response value
No Disease	O	0.0
Resistant	R	0.2
Resistant to Moderately Resistant	R-MR	0.3
Moderately Resistant	MR	0.4
Moderately Resistant to Moderately Susceptible	MR-MS	0.6
Moderately Susceptible	MS	0.8
Moderately Susceptible to Susceptible	MS-S	0.9
Susceptible	S	1.0

**Table 5:** Sequences (F/R) of SSR marker used for 10 yellow rust resistance genes

Gene	Marker	Sequence (5’-3’)	Size (bp)	Annealing	Reference
*Yr5*	STS7*	F: GTACAATTCACCTAGAGT	478 bp		[19]
	*STS8*	R: GCAAGTTTTCTCCCTAT		45	
*Yr9*	iag95*	F: CTCTGTGGATAGTTACTTGATCGA	1100 bp		[20]
		R: CCTAGAACATGCATGGCTGTTACA		55	
*Yr10*	*PSP3000*	F: GCAGACCTGTGTCATTGGTC	240 bp	52	[21]
	*PSP3000*	R: GATATAGTGGCAGCAGGATAC			
*Yr15*	*gwm11*	F: GGATAGTCAGACAATTCTTGTG	215 bp	52	[22]
		R: GTGAATTGTGTCTTGTATGCTTCC			
*Yr17*	*VENTRIUP*	F: AGG GGC TAC TGA CCA AGG CT	252 bp	45	[23]
	*LN2*	R: TGCAGCTACAGCAGTATGTACACAAAA			
*Yr18*	*csLV34*	F: GTTGGTTAAGACTGGTGATGG	150 bp	65	[24]
		R: TGCTTGCTATTGCTGAATAGT			
*Yr26*	we173*	F: GGGACAAGGGGAGTTGAAGC	259 bp		[25]
		R: GAGAGTTCCAAGCAGAACAC		*58*	
*Yr29*	*Xgwm-295*	F: AGGGAAAAGACATCTTTTTT	250 bp	56	[26]
		R: CGACCGACTTCGGGTTC			
*Yr30*	*Xgwm533*	AAGGCGAATCAAACGGAATA	120 bp	56	[27]
		GTTGCTTTAGGGGAAAAGCC			
*Yr36*	*Barc101*	F: GCTCCTCTCCACGATCACGCAAAG	175 bp	58	[28]
		R: GCGAGTCGATCACACTATGAGCCAATG			

*Marker types: STS marker

The relative area under the disease progress curve (rAUDPC) for each genotype was calculated by using the equation of Akello et al. [29], as follows: rAUDPC = (genotype AUDPC / susceptible genotype AUDPC × 100).

The Relative Resistance Index (RRI) was calculated using the country’s average relative percentage attack (CARPA), which was calculated according to Aslam [30] on a 0–9 scale, where 0 represents the most susceptible variety and 9 shows the most resistant variety. The standard for the desirable index was maintained at ≤ 7, whereas the value for the acceptable index was fixed using the following formula for the calculation of RRI [30, 31]:

$$\:RRI\:=\:(100\:-\:CARPA)\:/\:100\:\times\:\:9$$

### Marker-assisted selection and detection of selected stripe rust resistance genes

To transfer critical Yr genes, the Sakha Agriculture Research Station started a backcross program in 2021. Near-isogenic lines of “variety,” each expressing a unique *Yr* gene were crossed with wheat genotypes that reflected the necessary economic and technological advances in quality characteristics but were either vulnerable or moderately resistant to stripe rust. First-generation plants were backcrossed with recurrent parents. BC1 plants were chosen using marker-assisted selection (MAS) from different backcross generations, which were again backcrossed with the recurrent parent. Marker-assisted selection (MAS) involves the selection of agriculturally significant features for crop breeding using morphological and DNA markers that act as indirect selection requirements. In breeding programs, this procedure is used to increase the efficacy or efficiency of selection for the desired traits. MAS is a tool used for crop improvement across a range of crop species and traits. Despite this recognition, MAS helps improve the polygenic features. With the development of nested association mapping populations and recent advancements in marker technology, such as high-throughput genotyping of plants, MAS is expected that MAS will be easier, faster, cheaper, and more successful than traditional (phenotypic) selection. In addition, consider efficacy, the selection of Yr genes for integration also considers the availability of specific and closely associated PCR markers. These were used in BC generations that were segregated for Yr genes using molecular marker-assisted selection. Twenty wheat genotypes were designated to identify ten resistance genes (*Yr’s*): *Yr5*,* Yr9*,* Yr10*,* Yr15*,* Yr17*,* Yr18*,* Yr26*,* Yr29*,* Yr30*, and *Yr36* using specific primers for these genes, as listed in Table (5). The DNeasy^®^ Plant Mini Kit (Qiagen^®^) and the CTAB (cetyl-trimethy lammonium bromide) method (Rogers and Bendich ) [32] were used to isolate DNA from these twenty wheat genotypes.DNA was isolated from the leaves of five to seven-day-old seedlings. The PCR protocol was conducted at the EPCRS Excellence Center, Plant Pathology and Biotechnology Lab. (accredited with ISO/17025, ISO/9001, ISO/14001, and OHSAS/18001), Botany Department, Faculty of Agriculture, University of Kafrelsheik, Egypt. The PCR reaction mixture (15 µL) included a 5 ng DNA template, 10 pmol of forward primer, 10 pmol of reverse primer, 0.1 U of Taq DNA polymerase (Bioline GmbH, Germany), 25 mM MgCl_2_, 2 mM dNTPs, and 10× PCR buffer in 96-well thermal cyclers (Applied Biosystem Thermal Cycler, Singapore). The reaction conditions were as follows: initial denaturation for 5 min at 94^◦^C followed by 37 cycles of denaturation for 1 min at 94◦C, annealing for 1 min, and extension at 72 for 2 min. The annealing temperatures used are listed in Table (5). Then, a 10-minute final extension at 72◦C was completed. All PCR amplification products of SSR markers were separated by the electrophoresis on 2% agarose gels prepared in 1× TBE buffer and stained with ethidium bromide. A 100-bp DNA ladder was used as the DNA marker. The PCR products produced from *Yr26*, *Yr30*, and *Yr36* molecular marker analyses and disease reaction analyses were performed.

### Diploid program and back-crossing program

A diploid program was carried out to offer a unique opportunity to minimize the breeding cycle and fix agronomic features in the homozygous stage. Anthers in the mid-uninucleate phase were cultured in liquid MN6 induction medium [33]. The cultures were kept in the dark at 29 °C for 30 days, after which the embryogenic stage was transferred to a 190–2 regeneration culture comprising 0.09 M sucrose [34]. Plant regeneration was performed at 26 °C with a photoperiod of 16 h of light and 8 h of darkness. Green plantlets were transferred to test tubes containing hormone-free 190-2 regeneration cultures with 0.03 M sucrose and were vernalized for six weeks. Each haploid plantlet was treated with colchicine to the double chromosome to form a double–haploid plant in the test tubes, after which the wheat plants were cultured in the soil and raised until maturity. Six crosses produced by the diploid program were conducted between the *Yr5* and *Yr10* genes (male parents) and the three wheat cultivars Misr1, Misr2, and Gemmeiza11 (female parents) to obtain hybrid seeds. Seeds of the six F_1_ hybrid crosses were sown in one row, 2 m long, 30 cm apart, and 10 cm within rows, with four randomized complete block designs to have F_1_ seeds per cross. Seeds of each hybrid and F_1_ cross were planted in plots consisting of 12 rows (3 m in length and 30 cm apart). The plots were bordered by border rows of the highly sensitive variety Morocco, dusted by a mixture of uridinospore races and powder talcum (1:20) based on the approach of Tervet and Cassel [35] at early tillering. Disease severity % on F1 and F_2_ plants of each cross was recorded as the onset of yellow rust in any plant [16]. Frequency distribution values were calculated for yellow rust severity under field conditions for the parent, F_1_, and F_2_ populations.

### Thousand kernel weight

One way to measure seed size is to use the thousand-kernel weight (TKW). The grain yield of a wheat genotype (kg ha-1) is determined by its grain weight per unit area, or wheat grain yield, which is dependent on its seed value. One thousand grains of each type were randomly selected and weighed from each plot to determine the seed value.

### Cluster analysis

A similarity matrix of all wheat genotypes was created using phenotypic data, and molecular marker analysis was used to construct a dendrogram using the unweighted pair group method with the arithmetic means clustering method in the numerical taxonomy system (NTSYS-PC version 2.1).

### Statistical analysis

The correlation and regression coefficient “SPSS Regression Modeling” were used to determine the relationship between the evaluated wheat genotypes based on molecular marker analyses and phenotypic data over the three growing seasons of the study. Data analysis and final scores were only detected for the clear bands. For each marker, the band was scored as present (1) or absent (0) to create the binary dataset for the studied wheat genotypes. The genetic similarity coefficient was measured using the Dice coefficient. A dendrogram was created through cluster analysis using the unweighted pair group method of mathematics averages (UPGMA) for the exclusive marker systems. Three repetitions of a randomized complete block design (RCBD) were performed. Analysis of variance (ANOVA) was used to statistically examine the data using SPSS software V22.0 22 (SPSS Inc., Chicago, IL, USA). The least significant difference test was used to examine the differences between the parameters under study in the three seasons at the 95% confidence level (*p* = 0.05). To determine whether there were significant interactions between the independent variables and to look for significant changes between thousands of kernel weights for *Yr5* (A), *Yr10* (B), Misr1, Misr2, Gemmeiza11, as well as their F_1_ and F_2_ populations in the 2022 growing season, a one-way ANOVA and the LSD test were performed at the 5% level of confidence.

## Results

### Effectiveness of stripe rust resistance genes in Egypt

The effectiveness of stripe rust resistance genes has been determined by studying the field resistance of wheat genotypes to certain *Yr* genes for several decades. In this study, different resistance genes or alleles were propagated annually based on near-isogenic lines. The mean ACI values were assessed from stripe rust disease data recorded in artificially inoculated greenhouse in Egypt over the previous three years (2020, 2021, and 2022). The results demonstrated that 20 of the near-isogenic line (NIL) genotypes of wheat consuming a single *Yr* gene or allele are always not infected with the pathogen, or absolutely to a negligible extent. On average, over the previous three years, genotypes of wheat containing the genes *Yr5*,* Yr15*, *Yr37*, *Yr51* and *Yr57* established extraordinary resistance to stripe rust. The lines exhibiting the highest degree of infection were the NILs containing *Yr5* and *Yr15* genes.

### Evaluation of the wheat genotypes for stripe rust resistance

Stripe rust levels on thirty-eight wheat genotypes and the check variety were evaluated using four disease factors: ACI, AUDPC, rAUDPC, and RRI. The collected data showed that the three seasons’ differences in the virulence races of the wheat stripe rust pathogen and the minor variations in environmental variables caused the tested genotypes to vary in disease severity, which ranged from 1 to 80%. Based on the infection responses, the results showed that the wheat under study was split into three groups for each of the three seasons. The first group showed no response during the three seasons and was resistant to every tested isolate as presented in Tables 6 and 7, and 8. Misr3 and Misr4 are the genotypes of resistant. The second group displayed R-MR and MS-S wheat genotypes that have been reported. The remaining genotypes that were susceptible to S reactions were included in the third group.

**Table 6:** The six disease parameters for wheat genotypes of stripe rust in 2020 season

Genotypes	IT ^a^	ACI	AUDPC	rAUDPC	CARPA	RRI
**Race-specific** ^b^						
Misr3	NIR	0	0	0	0	9.00
Misr4	NIR	0	0	0	0	9.00
Giza 170	NIR	0	0	0	0	9.00
**Slow rusting I**						
Giza 171	RMR	1.5	07.5	0.44	1.67	8.85
Sakha 69	RMR	2.0	10.0	0.59	2.22	8.80
Gemmeiza 10	RMR	2.0	10.0	0.59	2.22	8.80
Sids 13	RMR	3.0	15.0	0.88	3.33	8.70
Sakha 93	MR	6.0	30.0	1.76	6.67	8.40
Sakha 94	MRMS	6.0	80.0	5.16	7.50	8.33
Giza 168	MRMS	8.0	60.0	3.87	10.00	8.10
Gemmeiza 9	MRMS	8.0	100.0	6.45	10.00	8.10
**Slow rusting II**						
Giza 162	MS	10	130	8.39	12.50	7.88
Giza 167	MS	10	150	9.68	12.50	7.88
Sakha 62	MSS	20	200	12.90	25.00	6.75
Sakha 95	MSS	24	200	11.76	26.67	6.60
Giza 164	MSS	27	215	12.65	30.00	6.30
Gemmeiza 5	MSS	20	225	13.24	22.22	7.00
Sids 1	MSS	36	340	20.00	40.00	5.40
**Susceptible**						
Sakha 61	S	20	255	15.00	22.22	7.00
Sakha 8	S	30	475	30.65	37.50	5.63
Sids 14	S	30	475	27.94	33.33	6.00
Sids 4	S	40	425	25.00	44.44	5.00
Sids12	S	40	500	30.00	44.44	5.00
Sids 8	S	40	500	30.00	44.44	5.00
Gemmeiza3	S	40	525	30.88	44.44	5.00
Sakha 92	S	50	575	33.82	55.56	4.00
Giza 163	S	50	675	39.70	55.56	4.00
Misr2	S	50	775	45.59	55.56	4.00
Sids 6	S	50	625	36.76	55.56	4.00
Shandweel 1	S	60	725	42.65	66.67	3.00
Misr1	S	60	825	48.53	66.67	3.00
Gemmeiza 11	S	60	850	50.00	66.67	3.00
Giza 155	S	70	975	57.35	77.78	1.00
Gemmeiza 1	S	70	975	57.35	77.78	2.00
Giza 150	S	70	1075	63.23	77.78	2.00
Giza 165	S	80	825	48.53	88.89	1.00
Giza 157	S	80	1125	66.18	88.89	1.00
Giza 160	S	80	1200	77.42	88.89	1.00
Check	S	90	1700	100.00	100.00	0.00
LSD 5%	-	0.258	64.687	0.016	-	-
1%	-	0.339	48.792	0.014	-	-

The average coefficient of infection (ACI), the area under the disease progress curve (AUDPC), The relative area under the disease progress curve (rAUDPC), Country Average Relative Percentage Attack (CARPA), The Relative Resistance Index (RRI) a ITs based on Roelfs et al., (1992)., 0 = Immune. R = resistant (necrosis with few uredinia); MR = moderately resistant (necrosis with small to moderate number of uredinia); MS = moderately susceptible (moderate number of uredinia with chlorotic areas); and S = susceptible (large number of uredinia, no necrosis but chlorosis may be evident); b Showing near immune reaction in all seasons at two growing seasons to *Pst*

**Table 7:** The six disease parameters of the 2021 season for wheat genotypes of stripe rust

Genotypes	FIT	ACI	AUDPC	rAUDPC	CARPA	RRI
Race-specific						
Misr3	NIR	0	0	0	0	9.00
Misr4	NIR	0	0	0	0	9.00
Giza 170	NIR	0	0	0	0	9.00
**Slow rusting I**						
Giza 171	RMR	0.6	03.0	0.19	0.75	8.93
Sakha 69	RMR	1.5	7.50	0.48	1.88	8.83
Gemmeiza 10	RMR	3.0	15.0	0.88	3.75	8.66
Sids 13	RMR	2.0	10.0	0.59	2.50	8.78
Sakha 93	RMR	1.0	05.0	0.32	1.25	8.89
Sakha 94	RMR	0.6	03.0	0.19	0.75	8.93
Giza 168	MR	4.0	20.0	1.29	5.00	8.55
**Slow rusting II**						
Giza 162	MS	8	60	3.87	10.00	8.10
Giza 167	MS	8	80	5.16	10.00	8.10
Sakha 62	MSS	16	160	9.41	17.78	7.40
Sakha 95	MSS	18	240	15.48	22.50	6.98
Sids 1	MSS	36	280	18.06	45.00	4.95
Sids 8	MSS	10	225	14.52	12.50	7.88
Sids 12	MSS	40	400	25.81	50.00	4.50
Gemmeiza 9	MSS	16	160	10.32	20.00	7.20
**Susceptible**						
Sakha 61	S	20	320	20.65	25.00	6.75
Sakha 8	S	20	425	27.42	25.00	6.75
Sids 14	S	40	450	29.03	50.00	4.50
Sids 4	S	30	400	25.81	37.50	5.63
Gemmeiza3	S	60	750	48.39	75.00	2.25
Sakha 92	S	40	525	33.87	50.00	4.50
Giza 163	S	40	600	38.71	50.00	4.50
Misr2	S	50	650	41.94	62.50	3.38
Sids 6	S	50	550	35.48	62.50	3.38
Shandweel 1	S	60	800	51.62	75.00	2.25
Misr1	S	60	900	58.06	75.00	2.25
Gemmeiza 11	S	60	925	59.68	75.00	2.25
Giza 155	S	60	700	45.16	75.00	2.25
Gemmeiza 1	S	70	950	61.29	87.50	1.13
Giza 150	S	70	850	54.84	87.50	1.13
Giza 165	S	70	800	51.62	87.50	1.13
Giza 157	S	80	900	58.06	100.00	0.00
Giza 160	S	40	600	38.71	50.00	4.50
Giza 164	S	70	900	58.06	87.50	1.13
Gemmeiza 5	S	20	200	12.90	25.00	6.75
Check	S	80	1550	100.00	100.00	0.00
LSD 5%	-	0.273	21.052	0.192	-	-
1%	-	0.206	15.882	0.147	-	-

The average coefficient of infection (ACI), the area under the disease progress curve (AUDPC), The relative area under the disease progress curve (rAUDPC), Country Average Relative Percentage Attack (CARPA), The Relative Resistance Index (RRI) b ITs based on Roelfs et al., (1992)., 0 = Immune. R = resistant (necrosis with few uredinia); MR = moderately resistant (necrosis with small to moderate number of uredinia); MS = moderately susceptible (moderate number of uredinia with chlorotic areas); and S = susceptible (large number of uredinia, no necrosis but chlorosis may be evident)

**Table 8:** The six disease parameters for wheat genotypes of stripe rust in 2022 season

Genotypes	FIT	ACI	AUDPC	rAUDPC	CARPA	RRI
**Race-specific**						
Misr3	NIR	0	0	0	0	9.00
Misr4	NIR	0	0	0	0	9.00
**Slow rusting I**						
Giza 171	RMR	1.5	19.5	1.03	1.76	8.84
Sakha 69	RMR	1.5	23.5	1.24	1.76	8.84
Gemmeiza 10	MR	8.0	112	5.89	8.89	8.20
Sids 13	RMR	3.0	40.5	2.13	3.33	8.70
Sakha 93	MR	4.0	58	3.05	4.44	8.60
Sakha 94	RMR	1.5	13.5	0.71	1.76	8.84
Giza 168	MR	8.0	125	6.58	8.89	8.20
Giza 170	MR	4.0	67	3.53	4.44	8.60
**Slow rusting II**						
Sakha 95	MSS	18	222	11.7	20.00	7.20
**Susceptible**						
Sakha 61	S	30	425	22.4	33.33	6.00
Sakha 8	S	30	415	21.8	33.33	6.00
Sids 14	S	60	1150	60.5	66.67	3.00
Sids 4	S	30	465	24.5	33.33	6.00
Sids 12	S	70	1400	73.7	77.78	2.00
Sids 8	S	50	950	50	55.55	4.00
Gemmeiza3	S	60	1225	64.5	66.67	3.00
Sakha 92	S	40	675	35.5	44.44	5.00
Giza 163	S	40	690	36.3	44.44	5.00
Misr2	S	60	1175	61.8	66.67	3.00
Sids 6	S	50	925	48.7	55.55	4.00
Shandweel 1	S	60	1240	65.3	66.67	3.00
Misr1	S	60	1200	63.2	66.67	3.00
Gemmeiza 11	S	80	1690	88.9	88.89	1.00
Giza 155	S	60	1150	60.5	66.67	3.00
Gemmeiza 1	S	70	1390	73.2	77.78	2.00
Giza 150	S	70	1500	78.9	77.78	2.00
Giza 165	S	70	1425	75	77.78	2.00
Giza 157	S	80	1700	89.5	88.89	1.00
Giza 160	S	40	675	35.5	44.44	5.00
Gemmeiza 5	S	30	465	24.5	33.33	6.00
Gemmeiza 9	S	30	475	**25**	33.33	6.00
Giza 162	S	80	1600	84.2	88.89	1.00
Giza 164	S	70	1350	71.1	77.78	2.00
Giza 167	S	70	1350	71.1	77.78	2.00
Sids 1	S	30	465	24.5	33.33	6.00
Sakha 62	S	30	462	24.3	33.33	6.00
Check	S	90	1900	100	100.00	0.00
LSD 5%	-	3.45	15.52	6.43	-	-
1%	-	2.21	10.23	4.23	-	-

The average coefficient of infection (ACI), the area under the disease progress curve (AUDPC), The relative area under the disease progress curve (rAUDPC), Country Average Relative Percentage Attack (CARPA), The Relative Resistance Index (RRI) b ITs based on Roelfs et al., (1992)., 0 = Immune. R = resistant (necrosis with few uredinia); MR = moderately resistant (necrosis with small to moderate number of uredinia); MS = moderately susceptible (moderate number of uredinia with chlorotic areas); and S = susceptible (large number of uredinia, no necrosis but chlorosis may be evident)

### Estimation of ACI values

The average coefficient of infection (ACI) was calculated by combining the disease severity (%) and infection type (IT). Wheat genotypes between 4 and 10 were moderately susceptible but genotypes with up to three ACI values indicated slight resistance. In contrast, low levels of resistance to adult plants were observed in wheat genotypes with ACI values greater than 10 (Tables 6 and 7, and 8). Each wheat genotype evaluated ranged from susceptible to moderately resistant genotypes. Misr3 and Misr4 showed no signs of stripe rust during any of the three seasons. These genotypes were thought to have durable resistance and were categorized as a highly resistant group of genotypes. In contrast, Giza170 was entirely resistant during the first and second seasons. The slow-rusting group was separated into two groups based on the infection type. The first one comprised eight genotypes: Giza171, Sakha69, Gemmeiza10, Sids13, Sakha93, Sakha94, and Giza168, Gemmeiza9, and exhibited ACI values ranging from 0.6 to 8.0 in the three seasons (Tables 6 and 7, and 8). The second group, comprising one genotype, displayed average coefficient of infection (ACI) values extending from 10 to 36 over the three seasons. The last group comprised the susceptible genotypes that had ACI values of up to 24, in comparison with the control genotype (80–90%) in the seasons.

### Estimated AUDPC, rAUDPC, and relative resistance index (RRI) values

The wheat genotypes under investigation could be categorized into three main groups depending on AUDPC values. The first group comprised wheat genotypes Misr3 and Misr4, which displayed zero AUDPC and rAUDPC values, and an RRI equal to 9. Therefore, they were categorized as an entirely resistant group of genotypes that exhibited race-specific resistance against the P. striiformis population during the three seasons. The second group contained genotypes with the lowest values of AUDPC and rAUDPC, less than 240 and 15.48%, respectively, and an RRI value of ≥ 7 ≤ 9 (resistant) in the three seasons. The final group comprised the fast-rusting genotypes, which included wheat genotypes exhibiting high AUDPC values and rAUDPC ratios extending by 1700 and 89.5%, respectively, compared to the check genotype, Morocco, which had the highest AUDPC value (1900) and the maximum rAUDPC ratio (100%) during the three seasons (Tables 6 and 7, and 8).

### Response of wheat genotypes carrying stripe rust

A diverse range of adult plant reactions under field conditions was observed in the studied wheat genotypes with identified stripe rust resistance genes. The final severity of stripe rust (%) for most of these genotypes varies widely from one year to another, influenced by minor changes in environmental conditions across the three seasons under investigation. The differential groups (Yr^,^s) and wheat genotypes with stripe rust resistance genes exhibited a broad spectrum of rust responses during the 2020, 2021, and 2022 seasons under field conditions (Table 9).

**Table 9:** Wheat genotypes (yr^,^s) at the adult stage responses to stripe rust during three growing seasons (2020, 2021 and 2022)

No.	Genotypes/gene	Season / Stripe rust responses		
		2020	2021	2022
1	Morocco	100 S	100 S	100 S
2	Avocet S - *YrA*	100 S	30 S	80 S
3	Avocet A + *YrA*	90 S	50 S	70 S
4	*Yr1*/6* AOC	30 S	20 S	70 S
5	SIETE CERROS T66	10 S	20 S	60 S
6	TATARA	0	TrS	0
7	*Yr5*/6* Avocet S	0	0	0
8	Yr6/6* Avocet S	100 S	60 S	70 S
9	Yr7/6* Avocet S	100 S	70 S	80 S
10	Yr8/6* Avocet S	5MR	0	0
11	Yr9/6* Avocet S	100 S	60 S	80 S
12	Yr10/6* Avocet S	0	0	10MS
13	Yr15/6* Avocet S	0	0	0
14	Yr17/ 6* Avocet S	10MS	30MSS	50MSS
15	Yr18/6* Avocet S	50MS	60MS	30MS
16	Yr24/6* Avocet S	30 S	30 S	30MSS
17	Yr26/6* Avocet S	50MS	30MS	60MRMS
18	Yr27/6* Avocet S	20MSS	10 S	20MRMS
19	YrSp/6* Avocet S	0	0	5MS
20	PAVON F 76	40 S	20 S	30 S
21	SERI M82	50 S	30 S	60 S
22	OPATA M 85	5MR	10MR	10MR
23	SUPER KAUZ	0	0	0
24	YrCv/6* Avocet S (*Yr32*)	50 S	30 S	60 S
25	PBW343	30 S	10 S	30 S
26	AOC-YR*3/3/ALTAR 84/AE.SQ//OPATA	50 S	30 S	30 S
27	AOC-YR*3//LALBMONO 1*4/PVN	70 S	40 S	60 S
28	AOC-YR*3 / PASTOR	90 S	70 S	100 S
29	POLLMER	90 S	30 S	30 S
30	PASTOR	20 S	40 S	60 S
31	REBECA F2000	60 S	30 S	50 S
32	FRANCOLIN #1	80 S	40 S	80 S
33	AOC-YR/QUAIU/#3	80 S	30 S	80 S
34	OPATA/PASTOR CO5607 A020	0	0	0
35	OPATA/PASTOR CO5607 A047	0	0	0
36	AOC-YR/QUAIU # 3	80 S	30 S	80 S
37	M10 (MUTATED C-306) / AOC-YR	50 S	10 S	80 S
38	CHUAN NONG 19	10R	10R	20MR
39	IRAGI	10MR	10MR	10MR
40	KOELZ W 11,192:AE	30MS	30MS	60MSS
41	PBW343/KKU	0	0	5 S
42	AOC-YR*3 //LALBMONO1*4/PVN	70 S	30 S	80 S
43	*Yr33*	0	0	10 S
44	*Yr34*	10MR	20MR	20MR
45	*Yr35 98M71*	60 S	30 S	70 S
46	*Yr37*	0	0	0
47	*Yr4BL*	10MR	20MR	20MR
48	*Yr51*	0	TrMR	5R
49	*Yr54*	50 S	50 S	80 S
50	*Yr57*	0	5MR	5R
51	*YrKK*	0	0	0
52	*YrAId*	5MR	5MR	0

% rust severity based on the modified Cobb Scale’s (Peterson et al., 1948) and response to infection as described by (Roelfs et al., 1992)

During the three seasons, genotypes carrying stripe rust, such as Super Kauz (*Yr9*) and Opata/Pastor, and resistance genes like *Yr5*,* Yr15*,* Yr33*,* Yr37*, and *Yrkk*, were completely resistant to stripe rust (no disease signs). Additionally, R-type or MR-type reactions were observed in certain genotypes (*Yr34*,* Yr51*,* Yr57*,* Yr4BL*, Opata 58, Chuan Nong 19, and IRAGI), which have shown resistance so far (Table 9). This study also revealed that the most significant resistance genes, *Yr1*,* Yr10*,* Yr32*, and *YrSp*, previously identified as resistant to the stripe rust races in Egypt (2020 and 2021), turned susceptible. This suggests that the virulence of the *P. striiformis* population in the 2022 season is constantly changing compared to the races in the two previous seasons.

### Changes of races in *P. Striiformis*, the wheat stripe rust pathogen

**Fig. 1:**
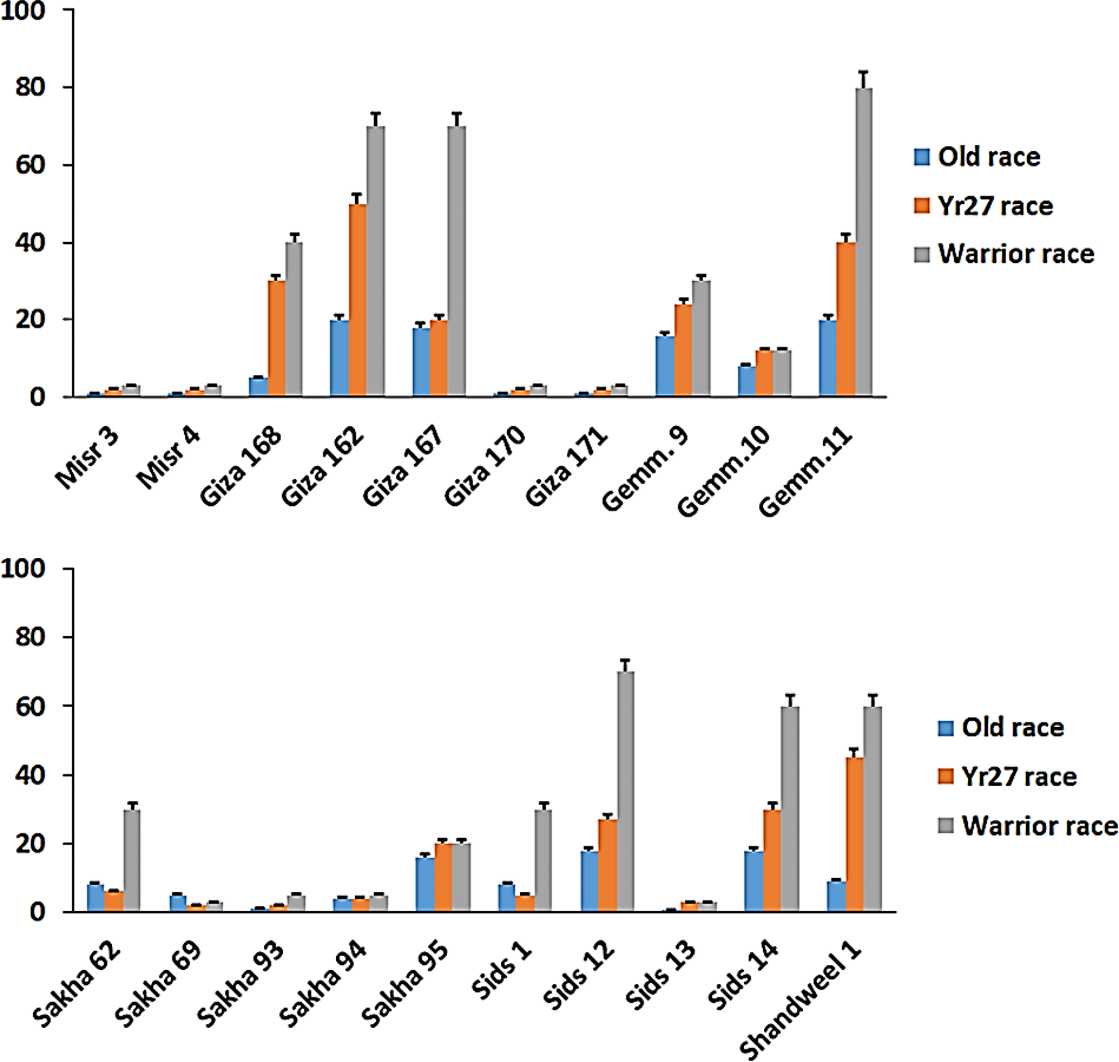
Stripe rust severity of 20 selected wheat genotypes inoculated with old races, *Yr27* race and warrior races at adult stage under greenhouse condition in 2022

Wheat stripe rust is a disease that thrives in maritime climate zones and affects most wheat-growing regions with cool and moist weather conditions, which are conducive to the widespread spread of the disease. The virulence of the stripe rust pathogen to the Yr genes in different genotypes leads to variations in races from year to year. New races, such as the Warrior race, emerged during the 2022 growing season and exhibited virulence towards wheat genotypes like *Yr10*,* YrSp*, and Giza170 (Fig. 1). This new race was aggressive towards *Yr1*,* Yr2*,* Yr3*,* Yr4*, *Yr6*,* Yr7*,* Yr9*,* Yr10*,* Yr17*,* Yr25*,* Yr32*,* YrSP*, and *YrAmb* (Table 3). In Egypt, older races (e.g., 0E0, 2E16, 6E4, 6E16, and 70E32) were virulent towards most wheat genotypes (Fig. 1). They exhibited aggressiveness towards certain genes like *Yr2*,* Yr6*,* Yr7*,* Yr8*,* Yr9*, and *Yr25* (Table 3). The *Yr27* race showed virulence towards *Yr2*,* Yr6*,* Yr7*,* Yr8*,* Yr9*,* Yr24*,* Yr25*, and *Yr27* (Table 3).

The new races (Warrior race) had a considerable change in the resistance level of many genotypes and arrived from Europe in 2011 (Fig. 1). Despite this, it was found that the genes *Yr5* and *Yr15* were resistant to all races. As for Misr 3 and Misr 4 genotypes, they remained resistant; however, several formerly resistant genotypes, such as Misr1, Misr2, and Giza165, became susceptible with ratings between 50 S and 60 S. Already susceptible genotypes, such as Sids12 and Gemmeiza11, became even more susceptible due to the higher aggressiveness of the new races.

### Molecular characterization of wheat genotypes

**Fig. 2:**
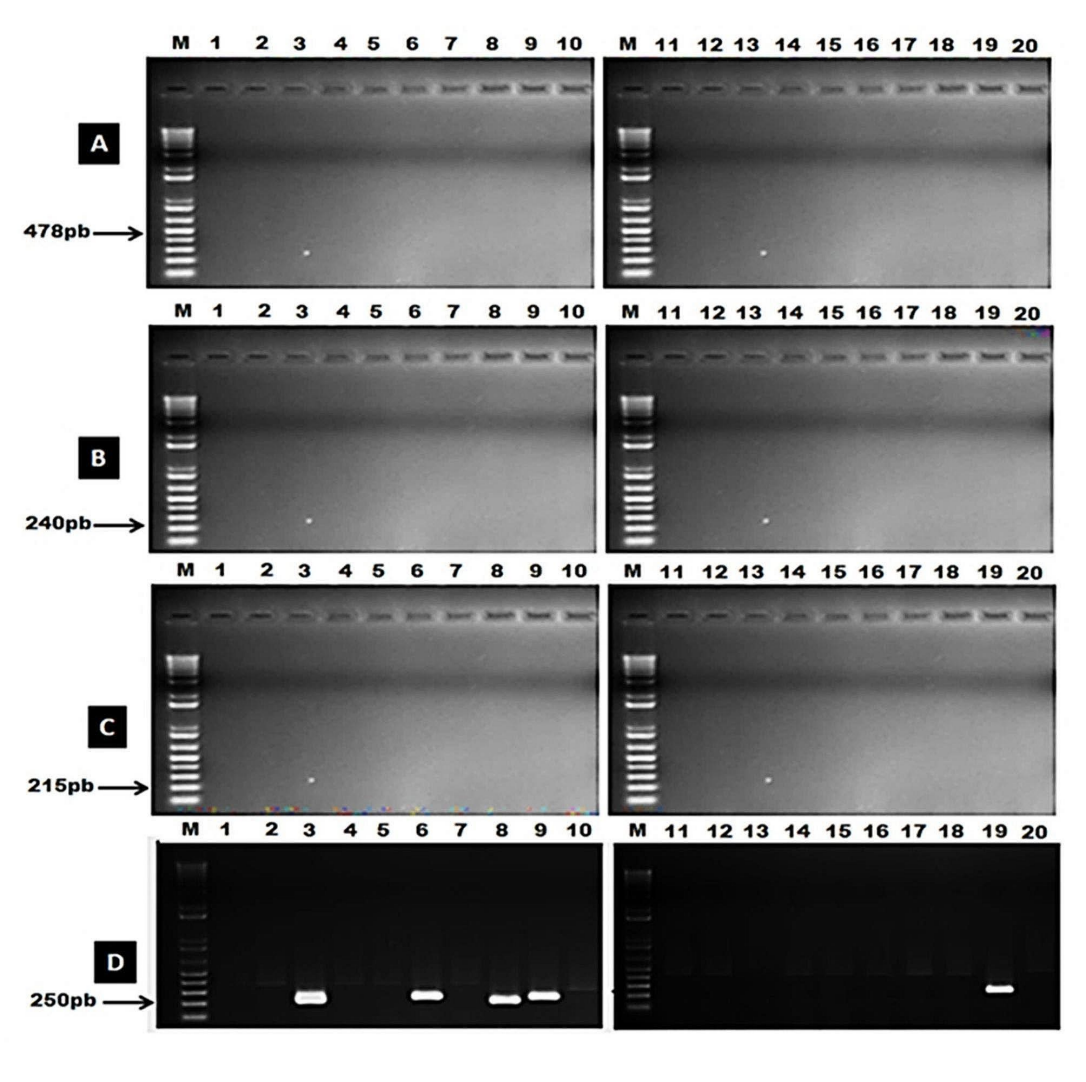
Amplification products of *Yr5***(A)**, *Yr10***(B)**, *Yr15***(C)**, and *Yr29* markers using PCR in the tested wheat genotypes running on agarose gel during the 2022 season. M: DNA Markar, Lane (1): Misr3, (2): Misr4, (3): Giza168, (4): Giza162, (5): Giza167, (6): Giza170, (7): Giza171, (8): Gemmeiza9, (9): Gemmeiza10, (10): Gemmeiza11, (11): Sakha62, (12): Sakha69, (13): Sakha93, (14): Sakha94, (15): Sakha95, (16): Sids1, (17): Sids12, (18): Sids13, (19): Sids14and (20): Shandweel 1

**Fig. 3:**
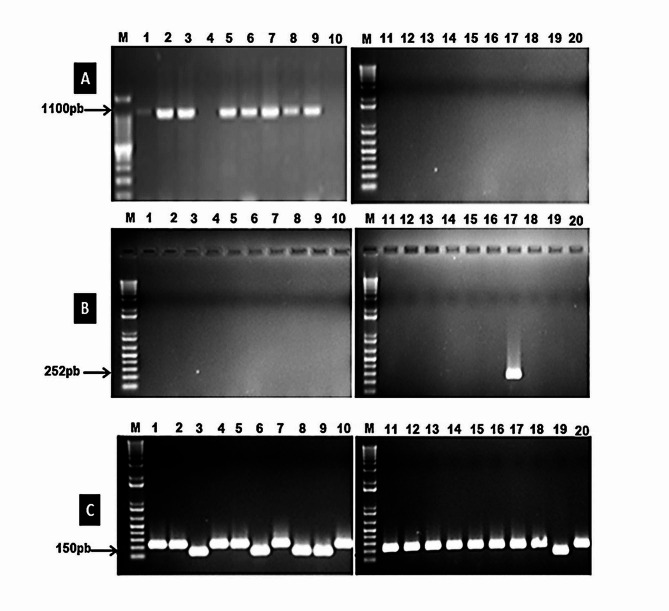
Amplification products of *Yr9***(A)**, *Yr17***(B)**, *Yr18***(C)** markers using PCR in the tested wheat genotypes running on agarose gel during the 2022 season. M: DNA Markar, Lane (1): Misr 3, (2): Misr 4, (3): Giza 168, (4): Giza 162, (5): Giza 167, (6): Giza 170, (7): Giza 171, (8): Gemmeiza 9, (9): Gemmeiza 10, (10): Gemmeiza 11, (11): Sakha 62, (12): Sakha 69, (13): Sakha 93, (14): Sakha 94, (15): Sakha 95, (16): Sids 1, (17): Sids 12, (18): Sids 13, (19): Sids 14 and (20): Shandweel 1

**Fig. 4:**
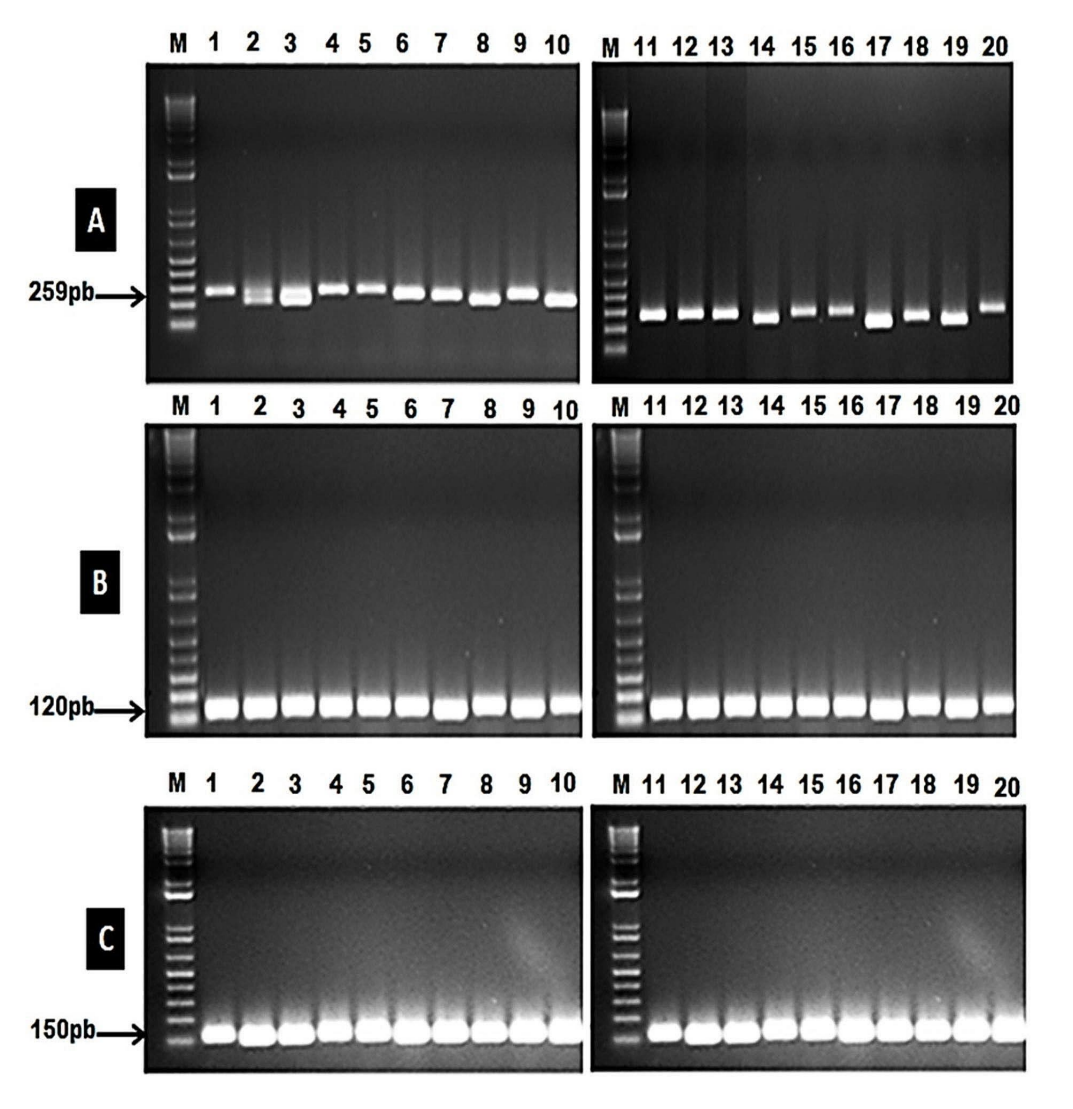
Amplification products of *Yr26***(A)**,* Yr30*** (B)** and *Yr36*** (C)** markers using PCR in the tested wheat genotypes running on agarose gel during the 2022 season. M: DNA Markar, Lane (1): Misr 3, (2): Misr 4, (3): Giza 168, (4): Giza 162, (5): Giza 167, (6): Giza 170, (7): Giza 171, (8): Gemmeiza 9, (9): Gemmeiza 10, (10): Gemmeiza 11, (11): Sakha 62, (12): Sakha 69, (13): Sakha 93, (14): Sakha 94, (15): Sakha 95, (16): Sids 1, (17): Sids 12, (18): Sids 13, (19): Sids 14, and (20): Shandweel 1

This investigation aimed to identify sources of resistance against *P. striiformis* populations in Egypt from 2020 to 2022. Wheat breeders now have essential information about stripe rust resistance genes to develop future breeding programs based on the molecular identification of effective resistance genes (Yr’s). The results revealed that *Yr5*,* Yr10*, and *Yr15* were not present in any wheat genotype (Fig. 2A, B, C; Table 10). However, *Yr29* gene was found in Giza 168, Giza170, Gemmeiza9, Gemmeiza10, and Sids14 (Fig. 2D; Table 10). On the other hand, the *Yr9* gene was only observed in eight genotypes (Misr3, Misr4, Giza168, Giza167, Giza170, Giza171, Gemmeiza9, and Gemmeiza10), while it was absent in the remaining genotypes (Fig. 3A; Table 10). The *Yr17* gene was detected in only one genotype, Sids 13 (Fig. 3B; Table 10). Additionally, the *Yr18* gene was found in Giza 168, Giza170, Gemmeiza-9, Gemmeiza-10, and Sids-14 (Fig. 3C; Table 10). Furthermore, *Yr26*,* Yr30*, and *Yr36* were identified in all genotypes under investigation (Fig. 4A, B, and C and Table 10). The markers *Yr5*,* Yr10*,* Yr15*, *Yr29*,* Yr9*,* Yr17*,* Yr18*,* Yr26*,* Yr30*, and *Yr36* were detected as fragments of 478, 240, 215, 250, 1100, 252, 150, 259, 120, and 150 bp in the positive control, respectively.

**Table 10:** Stripe rust resistance genes (*Yr’s*) detected by molecular marker analysis in twenty wheat genotypes

No.	Genotypes	Yr5	Yr9	Yr10	Yr15	Yr17	Yr18	Yr26	Yr29	Yr30	Yr36
1	Misr3	-	+	-	-	-	-	+	-	+	+
2	Misr4	-	+	-	-	-	-	+	-	+	+
3	Giza 168	-	+	-	-	-	+	+	+	+	+
4	Giza 162	-	-	-	-	-	-	+	-	+	+
5	Giza 167	-	+	-	-	-	-	+	-	+	+
6	Giza 170	-	+	-	-	-	+	+	+	+	+
7	Giza 171	-	+	-	-	-	-	+	-	+	+
8	Gemmeiza 9	-	+	-	-	-	+	+	+	+	+
9	Gemmeiza 10	-	+	-	-	-	+	+	+	+	+
10	Gemmeiza 11	-	-	-	-	-	-	+	-	+	+
11	Sakha 62	-	-	-	-	-	-	+	-	+	+
12	Sakha 69	-	-	-	-	-	-	+	-	+	+
13	Sakha 93	-	-	-	-	-	-	+	-	+	+
14	Sakha 94	-	-	-	-	-	+	+	-	+	+
15	Sakha 95	-	-	-	-	-	+	+	-	+	+
16	Sids 1	-	-	-	-	-	-	+	-	+	+
17	Sids 12	-	-	-	-	-	-	+	-	+	+
18	Sids 13	-	-	-	-	+	-	+	-	+	+
19	Sids 14	-	-	-	-	-	+	+	+	+	+
20	Shandweel 1	-	-	-	-	-	-	+	-	+	+

+= Fragment is amplified and the gene is present; - = No specific fragment is amplified and the gene is absent

### Genetic diversity analysis among wheat genotypes

**Fig. 5:**
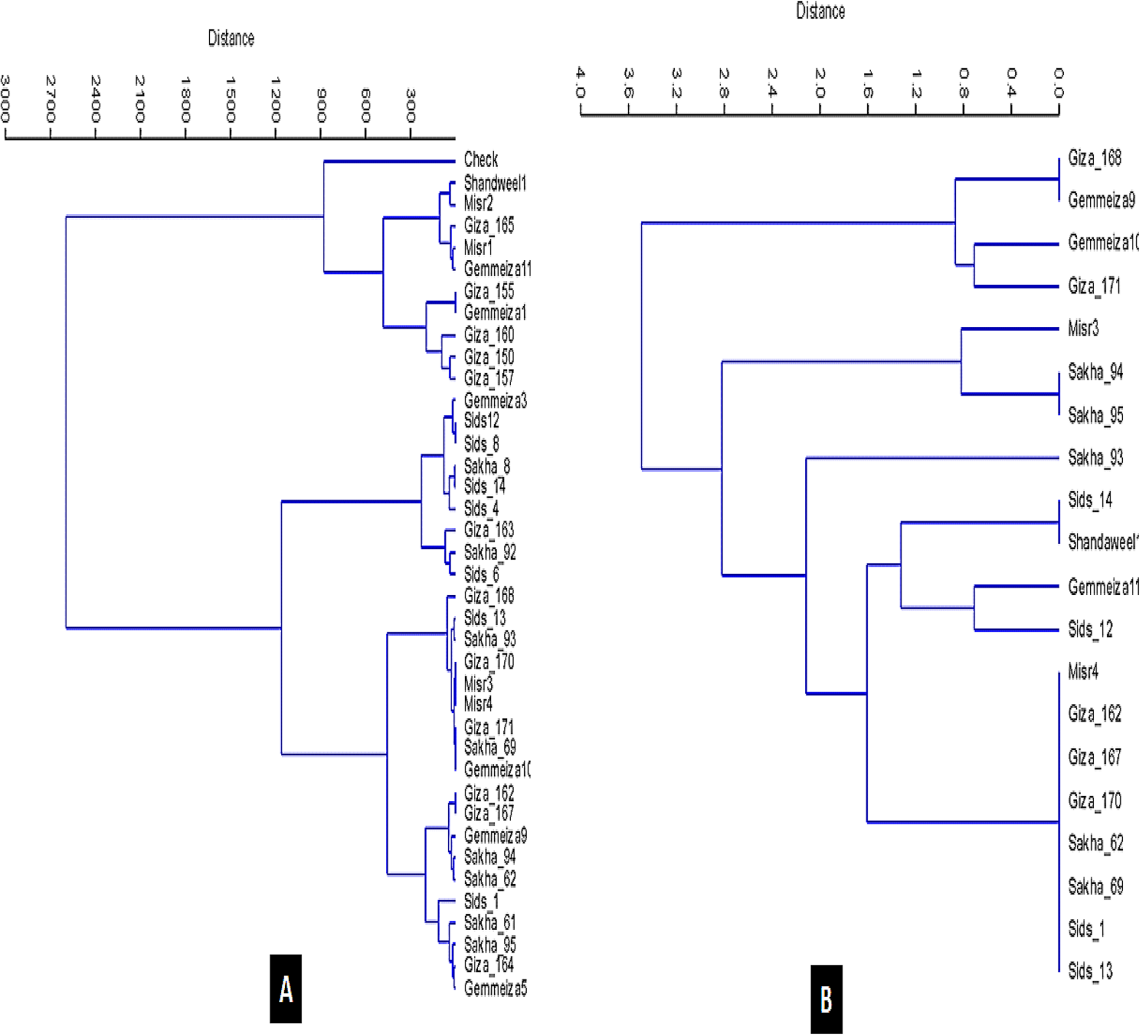
Dendrogram showing similarity index cluster analysis based on disease reaction **(A)** and molecular marker analysis **(B)** of wheat genotypes

Based on the genetic diversity analysis of slow rusting and molecular markers, wheat genotypes were categorized or clustered using rooted trees and the dendrogram. Based on slow rusting using rooted trees, Morocco was included as a separate line in the dendrogram (Fig. 5A) constructed based on banding pattern data, along with two groups for the evaluated lines. The first group was separated into two subgroups. The genotype Morocco was present in one subgroup, while the other subgroup encompassed Shandweel1, Misr2, Misr1, and Gemmeiza11 genotypes. High similarity was established between Misr1, Misr2 (susceptible) and Giza165 (susceptible). The second group was categorized into two subgroups; the first subgroup comprised Misr3, Misr4, and Giza170 (resistant) with the same similarity. These lines were similar depending on the three slow rusting parameters because these three genotypes were established to be immune, while the others included Sids13, Giza170, and Sakha 93 genotypes (moderately resistant). High similarity was detected between the Giza168 and Gemmeiza10 genotypes (resistant). The second subgroup consisted of the Sakha62 and Giza162 genotypes (moderately susceptible). Three groups were included in the dendrogram (Fig. 5B) created utilizing banding pattern data, which was based on the ten-stripe rust-specific markers for twenty wheat genotypes using rooted trees. The Giza171 and Gemmeiza10 genotypes were in a single, separate subgroup, so the first group has been divided into two. Giza168 and Gemmeiza9 genotypes with the same similarity value were found in the second subgroup.

### The marker-assisted backcross program and gene pyramiding

**Fig. 6:**
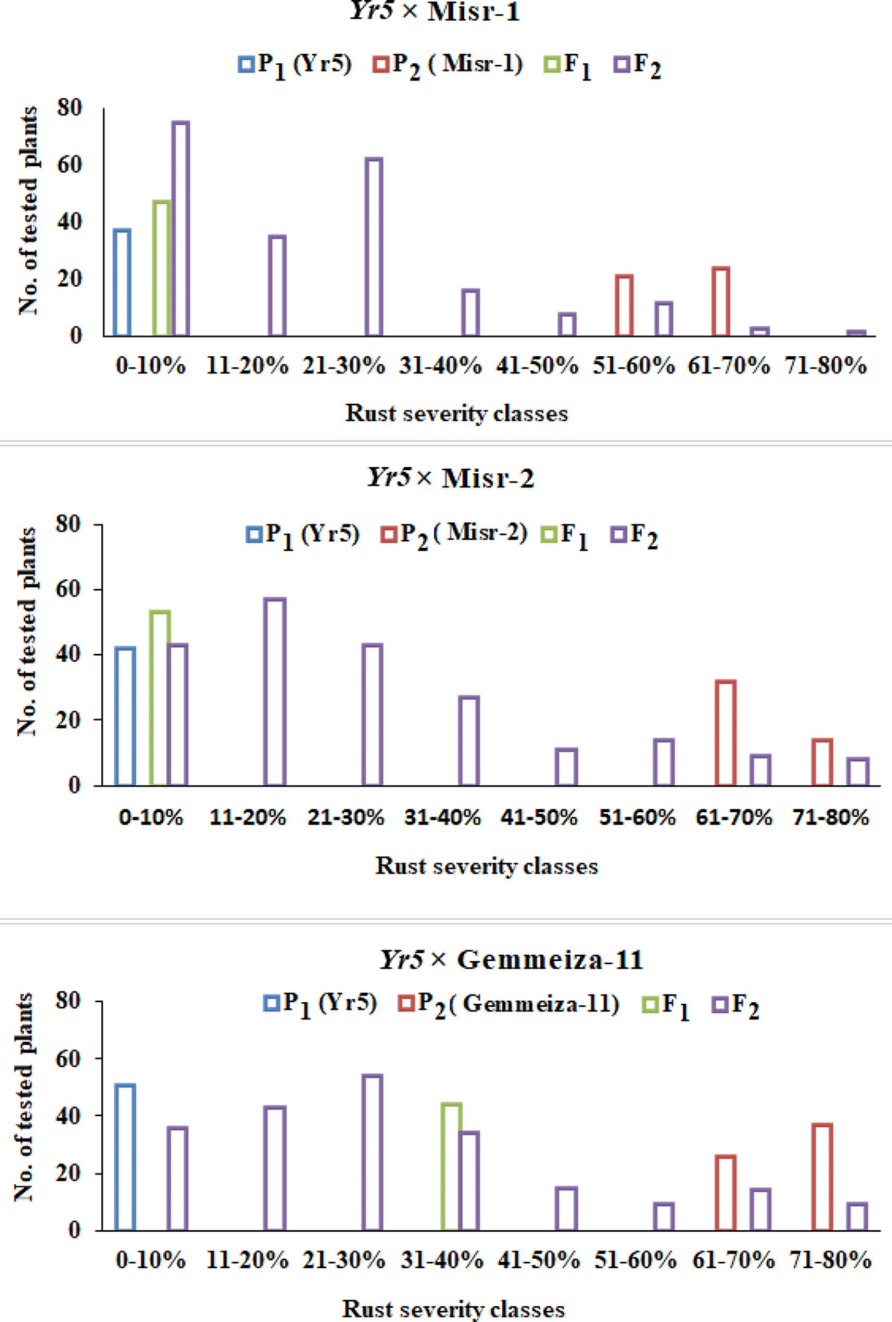
The frequency distribution of stripe rust severity (%) of three wheat crosses (P1, P2, F1 and F2) between *Yr5* and each of Misr-1, Misr-2 and Gemmeiza-11 inoculated with P. striiformis at adult stage

**Fig. 7:**
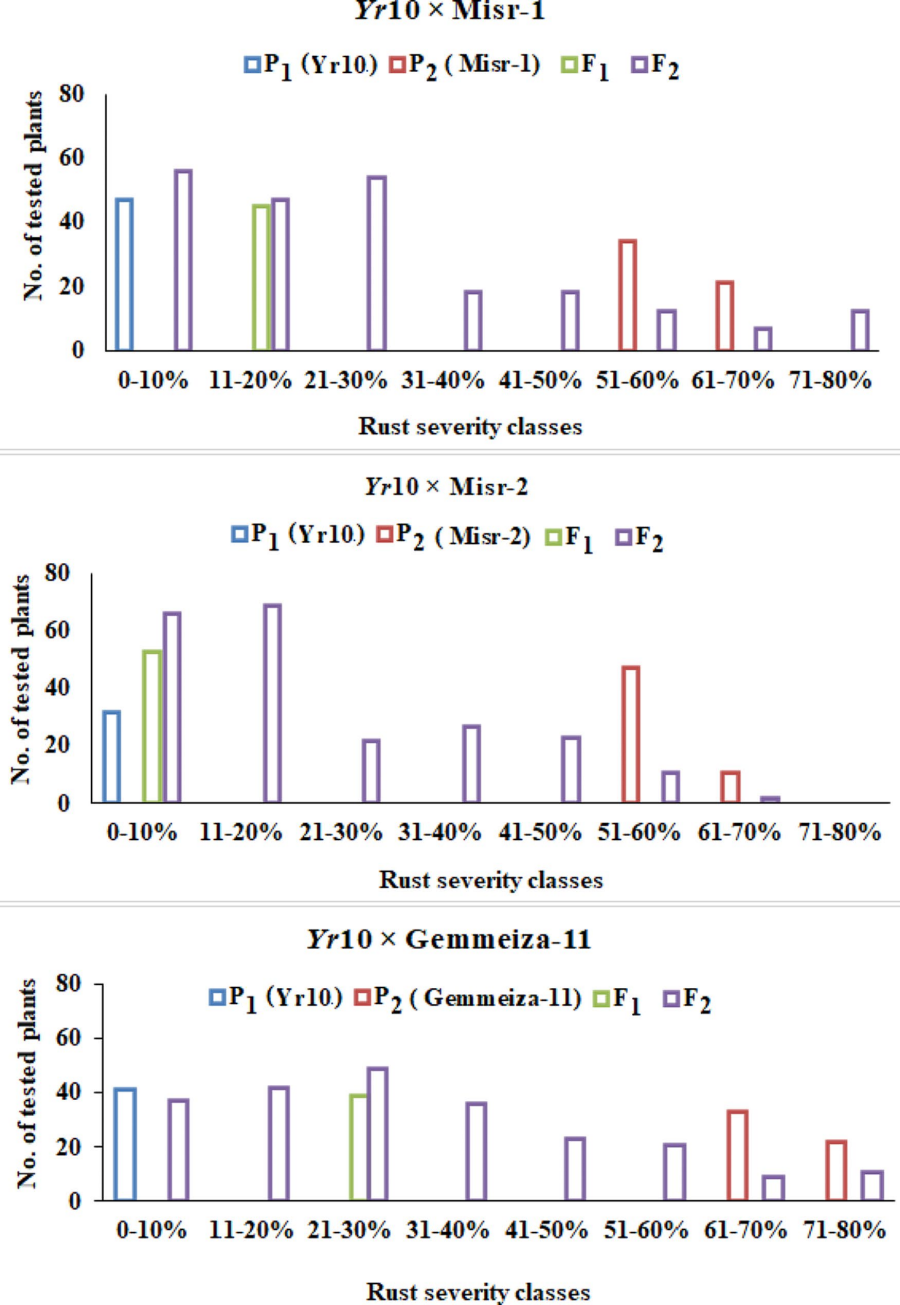
The frequency distribution of stripe rust severity (%) of three wheat crosses (P1, P2, F1 and F2) between Yr10 and each of Misr-1, Misr-2 and Gemmeiza-11 inoculated with P. striiformis at adult stage

**Fig. 8:**
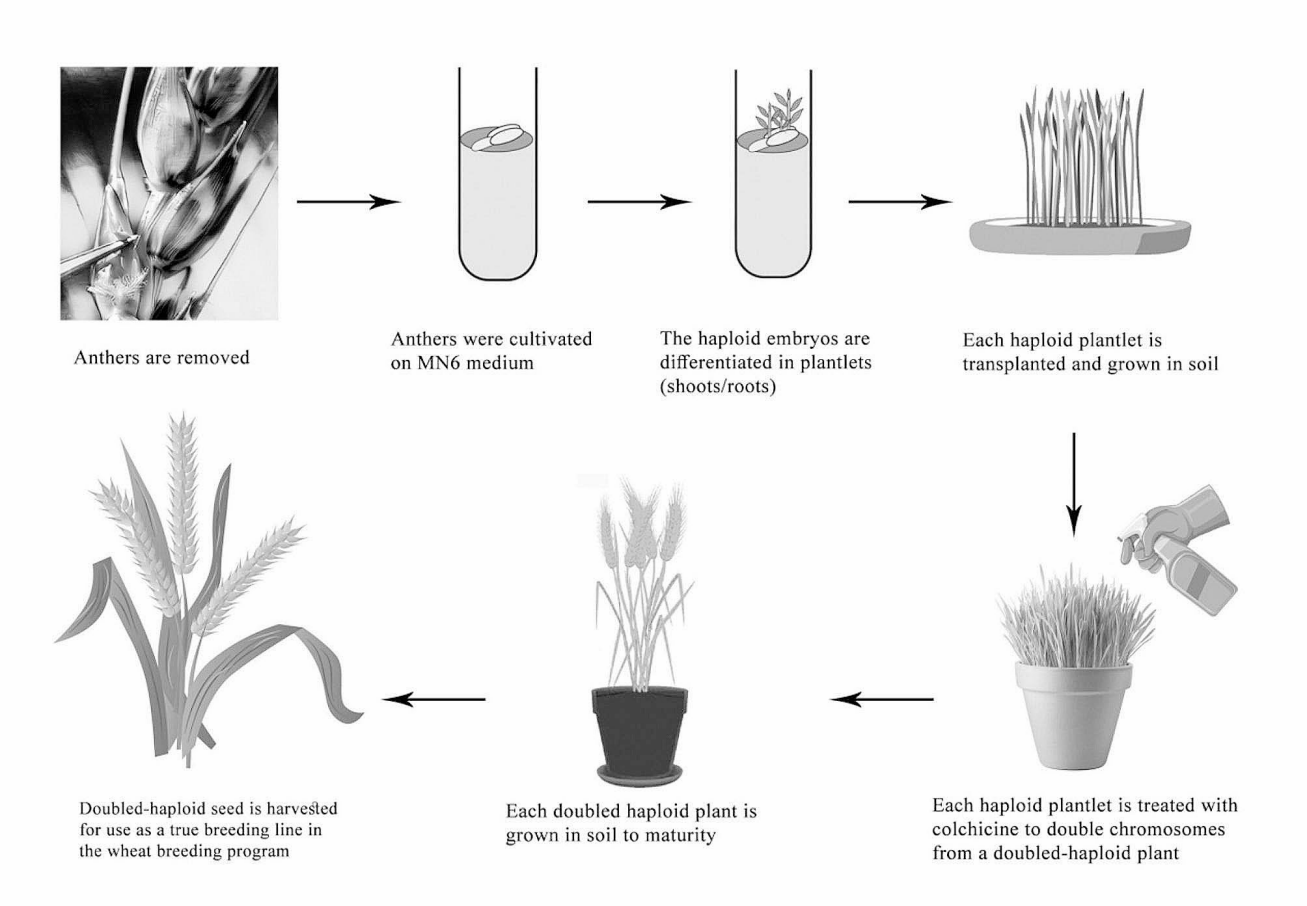
Doubled haploid breeding in wheat varieties in the tested wheat genotypes

A marker-assisted backcross program was established to introduce stripe rust resistance genes into three susceptible wheat genotypes (Misr1, Misr2, and Gemmeiza11). As depicted in Figs. 6 and 7, Misr1, Gemmeiza11, and Sids14 are susceptible to the pathogen. This study initially developed a marker-assisted backcross program to incorporate two genes providing adult resistance, *Yr5* and *Yr10*, by crossing susceptible wheat genotypes with resistant sources containing single Yr genes. The results demonstrated that the recurrent parents chosen (Misr1, Misr2, and Gemmeiza11) possess outstanding agronomic and qualitative traits; their resistance to Egypt stripe rust has been improved. Marker-assisted selection was implemented in the segregating generations. Currently, lines of the BC_2_ generation have been generated for various crosses (Figs. 6 and 7). Phenotypic analysis was utilized in each case to facilitate the marker-assisted selection of the Yr genes due to the unexplained link between the markers and the resistance genes. For the next generation of BC, only plants confirmed to carry the necessary resistance gene and exhibit resistance to stripe rust were utilized. To mitigate the risk of genetic vulnerability associated with using single yr genes, combinations of lines containing a variety of genes were considered to stack the genes. The underlying idea behind this research was that genotypes harboring multiple resistance genes simultaneously would exhibit greater resistance to stripe rust than those with only one gene. Currently, pyramided gene groups have been identified for the wheat cultivars. A double haploid program based on an alternative culture was established to preserve the gene combinations as illustrated in Fig. 8. The process of producing doubled haploid plants has commenced for most of the combinations. At this stage, plants with two stable Yr genes have been identified for specific combinations. Furthermore, the diploid program offers a new opportunity to reduce the breeding cycle and improve agronomic traits in the homozygous stage.

**Fig. 9:**
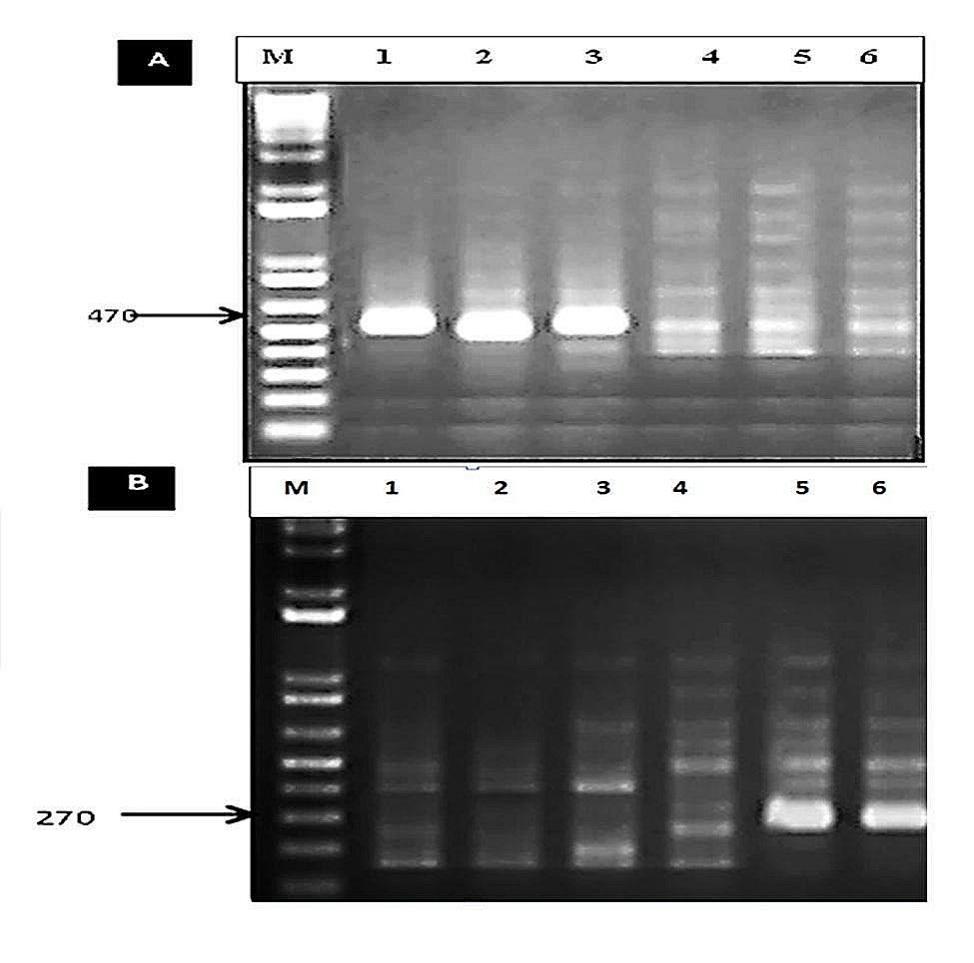
Amplification products of Yr5 **(A)** and Yr10 **(B)**, markers using PCR in the tested wheat genotypes running on agarose gel. **(A)** M: DNA Marker, Lane (1): Misr-1, (2): Misr-2, (3): Gemmeiza-11, (4): Yr5×Misr-1, (5): Yr5×Misr-2, (6): Yr5×Gemmeiza-11 and **(B)** M: DNA Marker, Lane (1): Misr-1, (2): Misr-2, (3): Gemmeiza-11, (4): Yr10×Misr-1, (5): Yr10×Misr-1, (6): Yr10×Gemmeiza-11

The second application of molecular markers in wheat genotype resistance breeding is the detection of specific resistance genes in genotypes for which the genetic background is unidentified. The presence of several genes is currently being examined in wheat genotypes and breeding lines developed in Egypt or used as parents in the breeding program. The results demonstrated the presence of a total of ten Yr genes (*Yr5*,* Yr9*,* Yr10*,* Yr15*,* Yr17*,* Yr18*,* Yr26*,* Yr29*,* Yr30*, and *Yr36*) in the twenty wheat genotypes studied in the breeding material. The Yr genes were also found in Egyptian breeding material through investigation. Misr1, Gemmeiza11, and Sids14 are among the identified types that are suspected to have the stripe rust resistance gene, which was also observed in several breeding lines as depicted in Fig. (9). In this context, fragments of 470 and 270 bp were detected for the markers *Yr5* (A) and *Yr10* in the positive control, respectively. The findings suggest that all of the selected genes may confer a significantly high level of resistance to the population.

### Wheat yield production

**Fig. 10:**
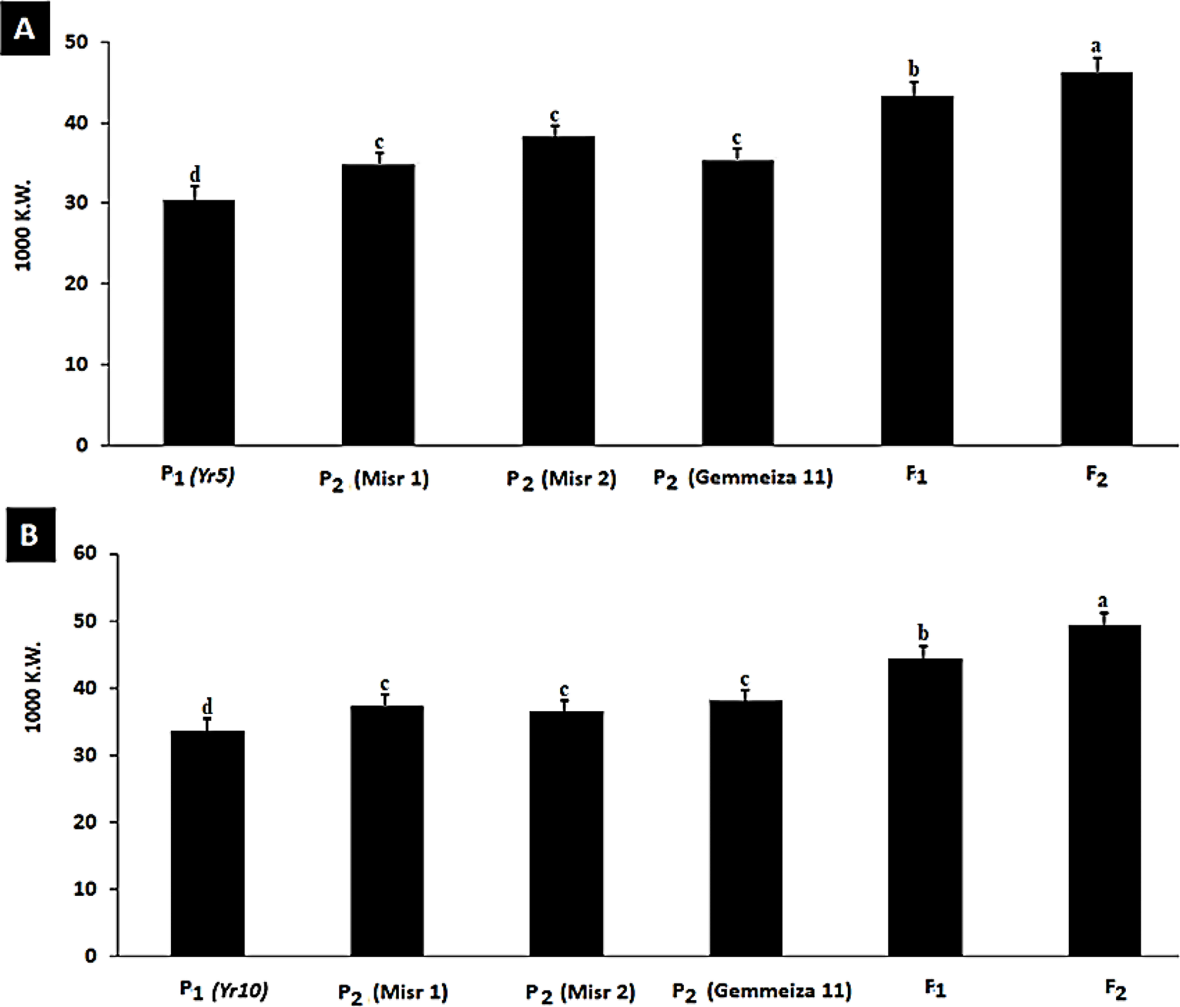
Thousands of kernel weight for *Yr5 (A)*,* Yr10 (B)*, Misr 1, Misr 2, Gemmeiza11, as well as their F1 and F2 populations in 2022 growing season

In this investigation, the results recorded in Table 11 results, which were obtained from this experiment, show that susceptible genotypes like variety Giza150 (24.04 g) had a significantly (P 0.05) lower seed value (46.9 g) than resistant genotypes like Misr 4. This suggests that, compared to susceptible genotypes (Giza150), the grains of resistant genotypes (Misr 4) are bolder and heavier. Similarly, the plots with bolder and larger seeds yielded heavier grains (average seed value of 40.80 g) compared to the less bold and smaller seed plots (10.24 g). The interaction between resistant genotypes and large-size seeds produced a maximum seed value of 46.9 g, according to the interactive effect data, while the interaction between susceptible genotypes and small-size seeds produced a minimum seed index value of 25.24 g. Genetics and seed size had a significant (P 0.05) impact on seed value, according to the analysis of letter variance shown in Figs. (9 and 10). However, their interacting effect was statistically non-significant (*P* > 0.05). The findings indicated that the quality of the seed directly affects the increase in seed value, and if farmers are aware of this and utilize high-value seed, they may achieve the desired grain weight. The findings indicated that the high quality of the seed directly affects the rise in seed value, and if farmers are aware of this and utilize high-value seed, they may achieve the desired grain weight. The breeding program’s results showed that susceptible genotypes, such as the Misr 1, Misr 2, and Gemmeiza 11 wheat varieties, had a significant impact on the seed value. Consequently, the results verified that the recurrent parents selected (Misr 1, Misr 2, and Gemmeiza 11) had exceptional agronomic and quality characteristics and produced large yields of wheat. Additionally, the F_1_ and F_2_ populations’ resistance to stripe rust in Egypt has improved susceptible wheat yields, as illustrated in Fig. (10).

**Table 11:** The seed index values of wheat varieties (1000 kernel weight) that were carried out

No.	Genotypes	2020	2021	2022
1	Misr1	34.3	35.1	30.2
2	Misr2	36.1	34.4	32.2
3	Misr3	45.3	44.2	45.4
4	Misr4	46.3	46.9	46.4
5	Sakha8	35.4	33.2	31.2
6	Sakha61	39.2	37.3	35.2
7	Sakha69	33.4	31.2	29.4
8	Sakha62	38.7	36.3	35.2
9	Sakha92	40.3	39.8	37.2
10	Sakha93	42.3	43.5	42.4
11	Sakha94	43.4	44.9	44.3
12	Sakha95	45.7	46.8	46.1
13	Gemmeiza1	29.8	27.3	26.5
14	Gemmeiza3	36.3	34.1	32.5
15	Gemmeiza5	39.8	37.3	35.4
16	Gemmeiza9	40.6	42.2	43.4
17	Gemmeiza10	41.3	42.4	43.2
18	Gemmeiza11	33.5	32.2	31.6
19	Giza150	27.6	26.2	24.4
20	Giza155	27.4	25.2	23.5
21	Giza157	27.7	25.9	24.3
22	Giza160	26.3	25.1	22.2
23	Giza162	40.2	42.5	43.8
24	Giza 163	29.3	28.6	26.2
25	Giza164	40.3	42.5	43.3
26	Giza165	27.6	26.4	24.2
27	Giza167	40.7	41.8	42.2
28	Giza168	41.3	42.4	43.2
29	Giza170	43.2	45.1	44.2
30	Giza171	46.5	46.9	45.4
31	Sids1	39.3	38.6	37.2
32	Sids4	40.3	41.7	40.0
33	Sids6	39.3	38.8	36.2
34	Sids8	38.6	37.2	36.4
35	Sids12	27.4	26.3	24.1
36	Sids13	42.2	44.2	43.4
37	Sids14	43.3	45.1	44.3
38	Shandweel1	41.3	42.2	40.6

## Discussion

*Puccinia striiformis* f. sp. *tritici*, the pathogen responsible for stripe rust, also known as yellow rust, is a significant threat to wheat (*Triticum aestivum*) in major wheat-growing regions worldwide [19, 20]. Nevertheless, creating genotypes of resistant wheat is the most economical means of managing the disease. This is considered the first line of defense and doesn’t cost the farmer anything extra. Relative resistance to wheat rust, especially stripe rust, is the main objective of most wheat breeding programs worldwide [21–24]. To achieve gene distribution, gene pyramiding, and the development of slow-rusting wheat genotypes, it is essential to identify genes for slow stripe rust resistance [25]. Likewise, there have been notable losses due to yellow rust, which has affected the common genotypes in multiple seasons. The discovery of sources of partial resistance was therefore essential [4].

Consequently, field evaluation of slow rusting resistance for thirty-eight wheat genotypes was performed through four disease characteristics: average coefficient of infection (ACI), area under the disease progress curve (AUDPC), relative area under the disease progress curve (rAUDPC), and relative resistance index (RRI). The data analysis revealed that the wheat genotypes under study exhibited diverse genetic backgrounds, leading to varying disease reactions to stripe rust. In wheat genotypes during the three studied growing seasons, the results revealed that three genotypes demonstrated an adequate level of near-immune resistance (NIR), twenty genotypes were susceptible (S), and five genotypes demonstrated a moderately resistant (R to MR) infection type, namely Giza171, Gemmeiza10, Sids13, and Sakha93. On the other hand, three genotypes demonstrated a moderately resistant to moderately susceptible (MR to MS) infection type, namely Sakha94, Giza168, and Gemmeiza9. Moreover, two genotypes demonstrated a moderately susceptible infection type, Giza 162 and Giza 167, while five genotypes demonstrated MS to S infection types, namely Sakha62, Sakha95, Giza164, Gemmeiza5, and Sids1. It may be inferred from the data that the two genotypes, Misr 3 and Misr 4, only showed a high degree of disease resistance and exhibited no outward symptoms of stripe rust infections or pustules. This kind of resistance to stripe rust could be attributed to a single main gene that functions well, with race-specific responses showing a near-immune reaction to stripe rust. Reliance on the slow-rusting genotype group is critical, particularly in severe situations (Sakha94, Giza171, Sids13, and Giza168). This outcome agreed with what Mabrouk 2022 [20] states.

Major resistance genes are often used in wheat resistance breeding, despite several drawbacks. The adoption of the marker-assisted selection has been made easier in recent years by improvements in molecular marker methods and marker documentation [7]. In this respect, marker-assisted selection in breeding programs is now possible through the recent advancements in molecular marker technologies and marker detection [7]. In the context of breeding wheat genotypes for resistance to stripe rust, it is mainly assumed that about sixty selected resistance genes and alleles that have been recognized using PCR-based markers can be used to distinguish between offspring populations.

To elucidate marker-assisted selection, 20 wheat genotypes were investigated in this study, and 10 specific primers, *Yr5*,* Yr9*,* Yr10*,* Yr15*,* Yr17*,* Yr18*,* Yr26*,* Yr29*,* Yr30*, and *Yr36*, were utilized to identify the genes responsible for resistance to yellow rust. Therefore, *Yr26*,* Yr30*, and *Yr36* were identified in every wheat genotype that was studied. It was determined that *Yr9* was present in 8 genotypes Misr3, Misr4, Giza168, Giza167, Giza170, Giza171, Gemmeiza9, and Gemmeiza-10 but not in the other genotypes. Several genotypes that were previously resistant, including Misr 3 and Misr 4, were no longer resistant, including Misr1 and Misr2, Sids12, and Gemmeiza11. Resistance genes like *Yr5*,* Yr10*, and *Yr15* were not identified in any genotypes under consideration, as evident from the study. Consequently, the Giza168, Giza170, Gemmeiza9, Gemmeiza10, and Sids14 genotypes were found to harbor the *Yr18* and *Yr29* genes. In this sense, genotypes carrying the Yr genes, such as Chuan Nong 19, Super Kauz, Opata/Pastor, Opata 58, and *Yr5*,* Yr15*,* Yr33*,* Yr37*,* Yrkk*,* Yr34*,* Yr51*,* Yr57*, and *Yr4BL*, were resistant to stripe rust during the three seasons [25, 26]. Changes in virulence in the *P. striiformis* population were indicated by the discovery that the most important resistance genes, *Yr1*,* Yr10*,* Yr32*, and *YrSp*, which were previously resistant to the previously identified races of stripe rust, were no longer effective. [2, 4]. The virulence of the strip rust infection in different genotypes and Yr genes determined how the race changed each year. [27] The newly established races developed and became virulent against wheat genotypes such as *Yr1*,* Yr10*,* Yr32*, and *YrSp*, as well as resistance genes. The warrior race that arrived from Europe in 2011 and the *Yr27* race were at their most virulent [28].

Current studies at CIMMYT have revealed that *Yr46* is closely linked to *Yr9* [36]. Gene *Yr46* is also closely linked to *Lr67* [37]. *Yr46* and *Lr67* genes confer slow rusting to yellow and leaf rusts [38]. Another minor gene, Yr30, included in the adult plant resistance of numerous CIMMYT wheat was detected to be in the chromosomal region containing the durable stem rust resistance gene *Sr2* [39]. On chromosome 7DS, the *Lr34/Yr18/Sr57/Pm38* complex was detected to confer adult-plant resistance (APR), slow rusting resistance, or partial resistance (PR) to leaf, yellow rusts, and several other diseases of wheat [25, 39]. The abrupt emergency as well as the quick and widespread proliferation of new hostile races led to the appearance of *PstS1* and *PstS2* in Egypt, which are continuously evolving in virulence. Its pathogenicity to *Yr1*,* Yr10*,* Yr27*,* Yr32*, and *YrSP* has sparked a search for novel sources of resistance to these aggressive races of wheat stripe rust [40].

Furthermore, DNA characterization of genotypes using ten specific primers was achieved to regulate the presence of the effective Yr resistance genes. Using a rooted tree dendrogram, the stowing of diversity and cluster analysis of wheat genotypes were performed. The studied wheat genotypes showed substantial genetic diversity. Moreover, Misr3, Attila, and PBW65” were closely associated with the wheat genotype in the pedigree (ATTILA*2/PBW65*2/KACHU) and considered a potential donor of Yr stripe rust resistance genes. Both parents’ genes for resistance to yellow rust. Also, wheat genotype Sakha94 with ‘OPATA’ in its pedigree is a possible donor of the *Yr18* stripe rust resistance gene.

The purpose of the breeding program is not to use lines carrying a single resistance gene as varieties. Consistent virulence has now been known for practically all the Yr genes in all the wheat-growing areas of the world [7], so if any line having a resistance gene that is still sufficient today was to be cultivated on a more extensive area, virulent pathotypes would soon reproduce in the pathogen population.

The intention is to construct sources with much superior agronomic qualities than the original donor kinds that were tailored to Egyptian conditions. To generate multiline variations from a combination of lines, or pyramid resistance genes, at the genotype level, near-isogenic lines expressing distinct Yr genes that were resulting from the same recurrent variety could be mated with each other [41, 42]. The “mix and match” process [43, 44], which compiles the line population that forms the multiline variation based on identical virulence, can be used to further improve the multiline idea in their instruction.

Experience to date suggests that markers flanking Yr genes can be used simply and effectively in marker-assisted backcross programs. However, the association between the markers and the resistance genes is not sufficient to select the most effective parental genotypes, requiring frequent phenotypic monitoring. The present results agree with those of Gyula et al. [44], as they noted that there is still a gap in the connection between the resistant Yr genes and the genetic markers. If appropriate parental genotypes are to be detected, then frequent phenotypic evaluation would be necessary. Resistance genes in wheat cultivars of uncertain ancestry can be readily documented with the aid of genetic markers. Programs for crossing can then be created using this knowledge. The breeding program was accelerated in this study by utilizing the benefits of the diploid program. Our results are consistent with those of Bambang et al. [40], as they demonstrated that another culture can accelerate upland rice breeding efforts.

Such research programs are significant because they could provide wheat breeders with information on stripe rust-resistant genes as well as ineffective genes that may be used to create stripe rust-resistant wheat genotypes. The present study also suggests that molecular markers should be rapidly and thoroughly utilized to analyze genetic diversity and explore the genetic basis of phenotypic variation in wheat for numerous accessions from a germplasm collection. Therefore, genetic diversity, where individuals within a population alter their alleles, often leads to the inheritance of these beneficial genetic traits from one generation to the next.

## Conclusion

The present investigation revealed significant differences in the number of resistance genes and their modes of action in wheat genotypes, as well as the final rust severity. *Yr5*,* Yr10*, and *Yr15* are identified as more effective and resistant genes in Egypt and worldwide against all races of *Pst*. In contrast, Gemmeiza11, Misr1, and Misr2 were susceptible to stripe rust due to not being recognized as resistance genes through marker-assisted selection and being widely cultivated by farmers. It is essential to enhance these genotypes by incorporating these resistance genes and reintroducing them. Therefore, it is recommended to utilize marker-assisted selection to identify resistant genes before initiating breeding programs to streamline the breeder’s tasks and accelerate the development of resistant varieties.

## Data Availability

The raw data will be available on request. Correspondence and requests for materials should be addressed to H.S.O.
